# Questionable research practices in ecology and evolution

**DOI:** 10.1371/journal.pone.0200303

**Published:** 2018-07-16

**Authors:** Hannah Fraser, Tim Parker, Shinichi Nakagawa, Ashley Barnett, Fiona Fidler

**Affiliations:** 1 School of BioSciences, University of Melbourne, Parkville, VIC, Australia; 2 Biology Department, Whitman College, Walla Walla, WA, United States of America; 3 School of Biological, Earth and Environmental Sciences, University of New South Wales, Sydney, NSW, Australia; 4 School of Historical and Philosophical Studies, University of Melbourne, Parkville, VIC, Australia; Tilburg University, NETHERLANDS

## Abstract

We surveyed 807 researchers (494 ecologists and 313 evolutionary biologists) about their use of Questionable Research Practices (QRPs), including cherry picking statistically significant results, *p* hacking, and hypothesising after the results are known (HARKing). We also asked them to estimate the proportion of their colleagues that use each of these QRPs. Several of the QRPs were prevalent within the ecology and evolution research community. Across the two groups, we found 64% of surveyed researchers reported they had at least once failed to report results because they were not statistically significant (cherry picking); 42% had collected more data after inspecting whether results were statistically significant (a form of *p* hacking) and 51% had reported an unexpected finding as though it had been hypothesised from the start (HARKing). Such practices have been directly implicated in the low rates of reproducible results uncovered by recent large scale replication studies in psychology and other disciplines. The rates of QRPs found in this study are comparable with the rates seen in psychology, indicating that the reproducibility problems discovered in psychology are also likely to be present in ecology and evolution.

## Introduction

All forms of science communication, including traditional journal articles, involve transforming complicated, often messy data into a coherent narrative form. O’Boyle et al [1] likened the process to a Chrysalis effect, turning “ugly initial results into beautiful articles”. Repeated failures to reproduce a large proportion of results in the published literature of other disciplines (e.g. [2,3]) has triggered reflection and meta-research about the ways in which this transformation process is susceptible to confusion and corruption. To date, large scale meta-research and replication projects have not been conducted in ecology and evolution [4,5]. However, many of the drivers of low reproducibility in other fields, such as publication bias and inflated type I errors, also appear common in ecology and evolution [6–9]. For example, Jennions and Møller [10] found that 38% of meta-analyses appeared to suffer publication bias, and that adjustments for missing (file drawer) studies changed the statistical conclusion (from statistically significant to non-significant) in 21% of cases. Low statistical power is a long-standing problem in ecology and evolution [11], and publishing only statistically significant studies from a pool with low average statistical power selects for inflated effect sizes and type I errors [12].

Forstmeier et al [11] further explain how, under the conditions of publication bias, Questionable Research Practices like *p* hacking and underpowered research can inflate number of false positive results in the literature. They offer a table of solutions for a range of problematic practices, all specifically relevant to research in ecology and evolution. The majority of advice concerns changes that individual researchers can make to improve the quality of their own research. However, some initiatives look to change the institutions and culture that influence individual behaviour by improving reporting standards in ecology and evolution journals [12,13] or switching the emphasis of research from p values to effect sizes [14,15]

The widespread prevalence of Questionable Research Practices (QRPs) is now well documented in psychology [16–18]. However, this is the first attempt (to the best of our knowledge) to document the prevalence of such practices in ecology and evolution.

### What are Questionable Research Practices (QRPs)?

QRPs refer to activities such as *p* hacking, cherry picking, and Hypothesizing After Results are Known (HARKing), all of which have been well documented in other fields including psychology and medicine. *Cherry picking* includes failing to report dependent or response variables or relationships that did not reach statistical significance or other threshold and/or failing to report conditions or treatments that did not reach statistical significance or other threshold. *P hacking* refers to a set of activities: checking the statistical significance of results before deciding whether to collect more data; stopping data collection early because results reached statistical significance; deciding whether to exclude data points (e.g., outliers) only after checking the impact on statistical significance and not reporting the impact of the data exclusion; adjusting statistical models, for instance by including or excluding covariates based on the resulting strength of the main effect of interest; and rounding of a *p* value to meet a statistical significance threshold (e.g., presenting 0.053 as *P* < .05). *HARKing* includes presenting ad hoc and/or unexpected findings as though they had been predicted all along [19]; and presenting exploratory work as though it was confirmatory hypothesis testing [20].

John et al [17] surveyed over 2000 psychological researchers in the US and asked about the prevalence of several questionable practices (we included questions about six of these practices in our survey, see Table 1). Agnoli et al [16] repeated John et al’s survey with a sample of Italian psychologists, and found strikingly similar results (also shown in Table 1). Failure to report outcome measures and stopping rules has also been documented by LeBel et al [21]. O’Boyle et al [1] found that in the process of translating PhD theses’ results to published articles the proportion of results supporting statistical hypotheses doubled; a change accounted for by the cherry picking of significant results.

**Table 1:** Journals used to identify researchers working in ecology and evolution.

Ecology Journals	Evolution Journals
Trends in Ecology and Evolution	Evolutionary Application
Ecology Letters	Evolution
Annual Review of Ecology and Evolution	BMC Evolutionary Biology
Frontiers in Ecology and the Environment	Evodevo
Global Change Biology	American Naturalist
Ecological Monographs	Journal of Evolutionary Biology
Methods in Ecology and Evolution	Evolutionary Biology
Journal of Ecology	Evolutionary Ecology
Global Ecology and Biogeography	Behavioural Ecology
ISME	
Journal of Applied Ecology	

### Publication bias and publish-or-perish research culture

Publication bias in this context refers to a bias towards publishing statistically significant, ‘positive’ results and not publishing statistically non-significant (‘negative’ or null results). The bias exists in many sciences [22], has been documented for decades in some disciplines (e.g., in psychology, see Sterling, 1959 [23]) and may be getting stronger across science, with a detectable increase in the proportion statistically significant results over the last 25 years [24].

The intersection of increasing publication bias and a growing publish-or-perish culture in science may well impact the frequency with which researchers employ QRPs [16,25]. In a publish-or-perish research culture, studies that were once relegated to a file drawer upon failing to reach statistical significance may now be more likely to be cherry picked, *p* hacked and HARKed back into the literature. In a simulation study, Smaldion & McElreath [26] demonstrate how selection for higher output can speed up the dissemination of poor methods within a research community.

Simmons et al [27] used simulated experimental data to demonstrated how QRPs such as reporting only the subset of dependent/response/outcome variables or experimental conditions that reached statistical significance can inflate the false positive error rate of the research literature. They warned of ‘researcher degrees of freedom’ in experimental reports, including failing to report the sampling stopping rule. This has been further demonstrated in an ecology and evolution context by Forstmeier et al [11]. QRPs, due to their propensity to increase the false positive rate, have been implicated as a contributing factor to the well-publicised reproducibility crisis in psychology and other disciplines [2,25,28].

### Aims

Publication bias in a publish-or-perish research culture incentivises researchers to engage in QRPs, which inflate the false positive rate leading to a less reproducible research literature. In this sense, QRP rates might be indicators of future reproducibility problems. Arguments about the difficulties in directly evaluating the reproducibility of the ecology and evolution literature have been made elsewhere (e.g., Schnitzer & Carson [29] but see Nakagawa & Parker [4]). However, the link between QRPs and irreproducibility is rooted in fundamental statistical theory [30] and so even in the absence of direct replication measures, a high prevalence of QRPs should alone raise sufficient concern to trigger editorial and institutional action.

The specific aims of our research were to:

1. Survey ecology and evolution researchers’ own self-reported rate of QRP frequency
2. Survey ecology and evolution researchers’ estimated rate of QRP use in their field
3. Compare these rates to those found in other disciplines, particularly psychology, where serious reproducibility problems have been established
4. Explore, through researchers’ open-ended comments on each QRP in the survey, attitudes, (mis)understandings, pressures and contexts contributing to QRP use in the discipline

## Methods

### Survey participants

We collected the email addresses of corresponding authors from 11 ‘ecology’ and 9 ‘evolutionary biology’ journals (see Table 1) in line with ethics approval supplied the University of Melbourne Human Research Ethics Committee (Ethics ID: 1646917.1). Journals were chosen from the highest ranking (assessed by 5-year impact factor) journals within the categories defined by the ISI 2013 Journal Citation Reports [31]. From the highest impact journals, we selected those that publish a broad range of work and excluded those limited to narrower sub-fields.

We extracted authors’ email addresses articles published in ecology journal (first 10 ecology journals listed in Table 1) issues between January 2014 and May 2016. We began a trial release of the survey (to check for bugs) on the 5^th^ of December 2016, we had sent the survey to all authors of articles in ecology journals by the 6^th^ of March 2016.

Before we looked at the initial data, we decided to expand our sample to include evolutionary biology researchers, and add authors from articles from the Journal of Applied Ecology. We collated email addresses from authors of articles in evolutionary biology journal (Table 1) issues and Journal of Applied Ecology issues between January 2015 and March 2017. We sent the email to these new participants on the 19^th^ of May 2017.

We deduplicated our list of email addresses before we sent each survey out to ensure that individual researchers did not receive our survey more than once. We ultimately emailed a total 5386 researchers with a link to our online survey which returned 807 responses (response rate = 15%).

Of the 807 responses, 71% (n = 573) were identified through our ‘ecology’ journal sample and 37% (n = 299) from our ‘evolution’ journal sample. This imbalance is a product of the number of journals in each sample and the order in which email addresses were collected and deduplicated; we first targeted ecology journals, and then decided to add a second group of evolution journals. Recognising that journal classification is only an approximate guide to disciplinary status, we asked researchers to self-identify their discipline; 411 researchers completed this question. Based on this information we made some adjustments to disciplinary classification as follows. First, we classified responses associated with sub-disciplines including the following terms as being made by evolution researchers: ‘evolut*’, ‘behav*’, ‘reproductive’, or ‘sexual’. From the remaining set of descriptions, we classified all responses associated including the following terms as being made by ecology researchers: ‘plant’, ‘*population’, ‘marine biology’, ‘biodiversity’, ‘community’, ‘environment*’, ‘conservation’, ‘ecology’, ‘botany’, ‘mycology’, or ‘zoology’. Researchers who did not use any of these terms and those who did not complete the self-identified sub-discipline question (n = 396) were left in their original journal discipline category as outlined in Table 1. At the end of this reclassification process, the sample (n = 807) consisted of 61% (n = 494) ecology researchers and 39% (n = 313) evolution researchers.

Only 69% (558-560/807) of our sample completed the demographic questions at the end of our survey. Of the 560 who completed the gender question, 69% identified as male, 29% as female, 0.2% identified as non-binary and 1% preferred not to say. Of the 558 who completed the career status question, 6% identified as graduate students, 33% as post-doctoral researchers, 24% as midcareer researchers/academics and 37% as senior researchers/academics. The 559 who completed the age question were divided between age categories as follows: under 30 (11.5%), 30–39 (46.7%), 40–49 (25.9%), 50–59 (9.8%), 60–69 (4.8%), and over 70 (1.3%).

### Survey instrument

Our research practices survey was administered via Qualtrics (Provo, UT, USA).The survey (S1 Supplementary Material) included questions about the following ten research practices:

1. Not reporting studies or variables that failed to reach statistical significance (e.g. p ≤0.05) or some other desired statistical threshold.
2. Not reporting covariates that failed to reach statistical significance (e.g. p ≤0.05) or some other desired statistical threshold.
3. Reporting an unexpected finding or a result from exploratory analysis as having been predicted from the start.
4. Reporting a set of statistical models as the complete tested set when other candidate models were also tested.
5. Rounding-off a p value or other quantity to meet a pre-specified threshold (e.g., reporting p = 0.054 as p = 0.05 or p = 0.013 as p = 0.01).
6. Deciding to exclude data points after first checking the impact on statistical significance (e.g. p ≤ 0.05) or some other desired statistical threshold.
7. Collecting more data for a study after first inspecting whether the results are statistically significant (e.g. p ≤ 0.05).
8. Changing to another type of statistical analysis after the analysis initially chosen failed to reach statistical significance (e.g. p ≤ 0.05) or some other desired statistical threshold.
9. Not disclosing known problems in the method and analysis, or problems with the data quality, that potentially impact conclusions.
10. Filling in missing data points without identifying those data as simulated.

Questions 1 to 9 were shown in random order but question 10 was always shown last, because it is particularly controversial and we did not want it to influence the responses to other items. For each of these 10 practices, researchers were asked to:

- estimate the percentage of ecology (evolution) researchers who they believe have engaged in this practice on at least one occasion (0–100%)
- specify how often they had themselves engaged in the practice (never, once, occasionally, frequently, almost always)
- specify how often they believe the practice *should* be used (never, rarely, often, almost always)

At the end of each QRP, researchers had the opportunity to make additional comments under the open-ended question: ‘why do you think this practice should or shouldn’t be used?’.

At the end of the set of 10 QRP questions, researchers were asked “have you ever had doubts about the scientific integrity of researchers in ecology (evolution)?”, and asked to specify the frequency of such doubts, if any, for different sub-groups. Finally, the survey included demographic questions about participants’ career stage, gender, age and sub-discipline, discussed above.

### Data analysis

**Fig 1:**
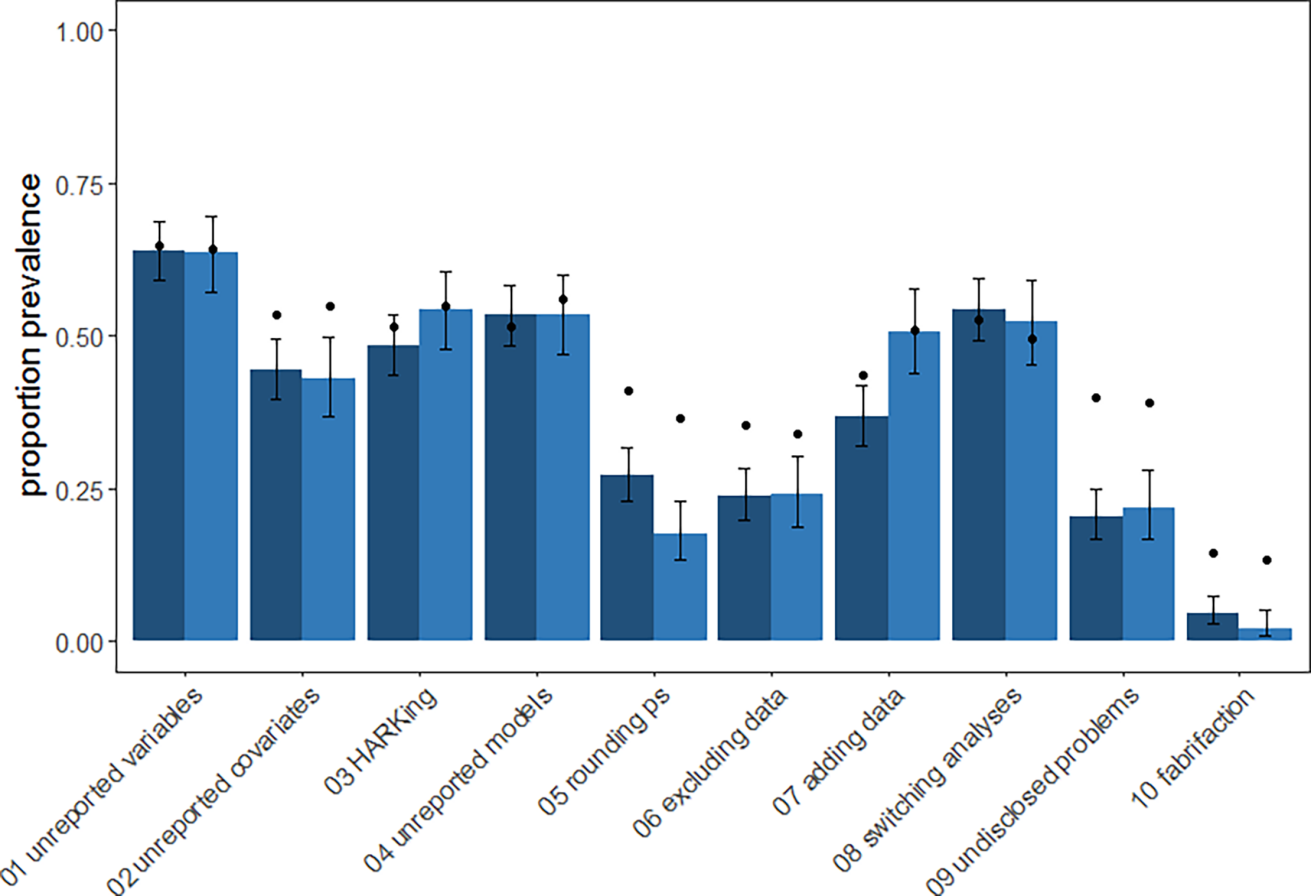
The prevalence of Questionable Research Practices in ecology and evolution. Light columns represent the proportion of evolution researchers and dark columns represent the proportion of ecology researchers who reported having used a practice at least once. The dots show researchers’ mean estimates of suspected use by colleagues in their field. Dots that are much higher than bars may suggest that the QRP is considered particularly socially unacceptable [17]. Error bars are 95% confidence intervals.

**Fig 2:**
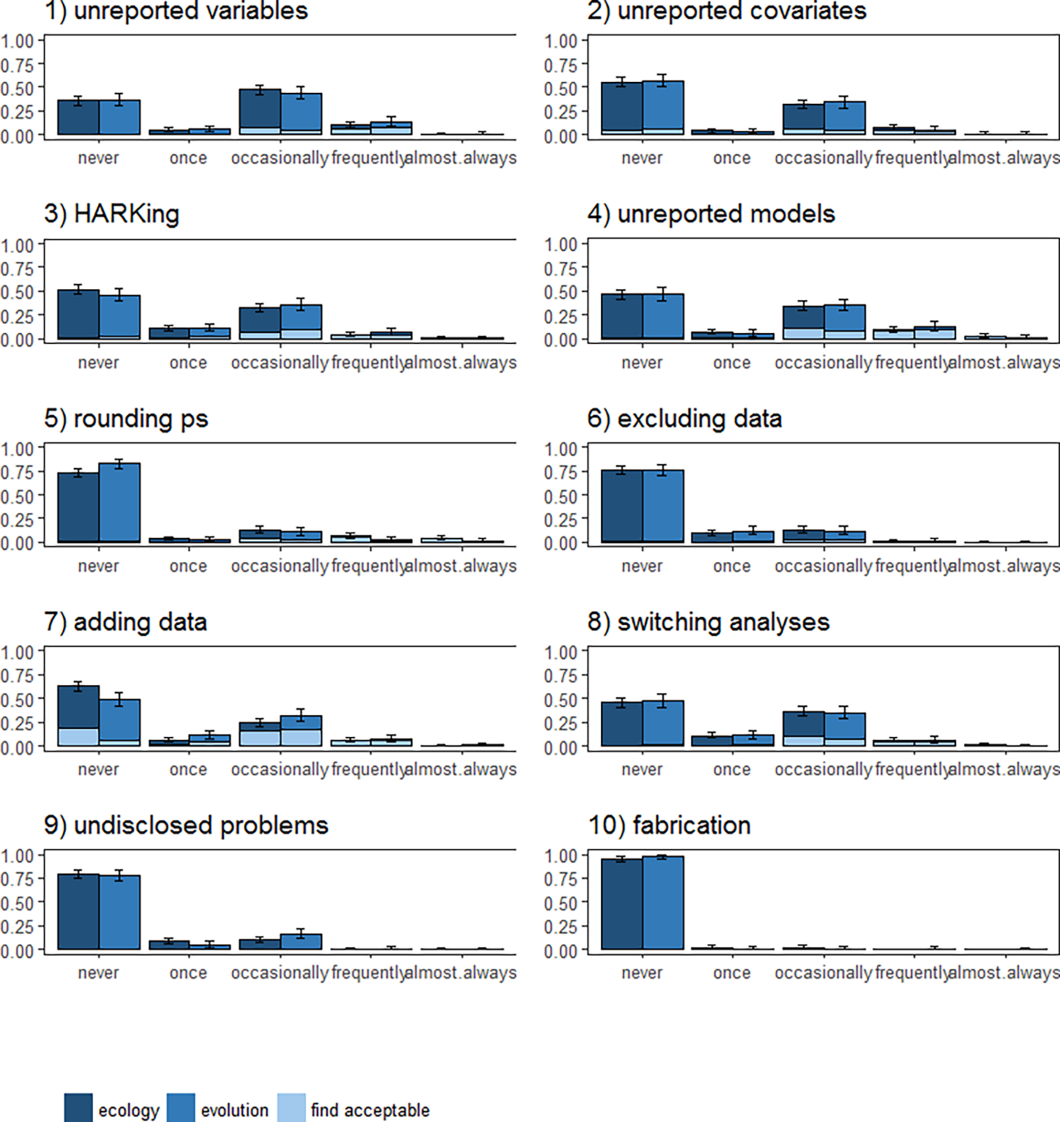
Proportion of researchers in ecology and evolution reporting frequency of use (or not) of 10 Questionable Research Practices. Shading indicates the proportion of each use category that identified the practice as acceptable. Error bars are 95% confidence intervals.

Analyses were preregistered after data collection had commenced but before the data were viewed [32] and performed in R version 3.3.3 [33]. The code and data required to reproduce our results are available from https://osf.io/qxt3u/. In Fig 1 we plotted the proportion of researchers reporting that they had used each of the 10 QRPs at least once against the researchers’ estimates of prevalence in the field, i.e., researchers’ responses to question (i) above. For each of the 10 QRPs we also plotted the proportion (with 95% Confidence Intervals, CIs) of researchers in each discipline who stated that they had used the practice ‘never’, ‘once’, ‘occasionally’, ‘frequently’, and ‘almost always’ in response to question (ii) above using *ggplot2* [34] (Fig 2). For the QRPs also covered in the John et al [17] and Agnoli et al [16] surveys, we directly compared proportions of researchers who had engaged in each QRP at least once (Table 2), as this is the primary frequency measure reported in those articles. We examined correlations between how frequently each participant had engaged in a practice and how acceptable they found the practice, and their age and career stage using Kendall’s Tau correlation. All 95% CIs are Wilson Score Intervals except for those on Kendall’s Tau, which are bootstrapped based on 1000 bootstrapped samples using *NSM3* [35].

**Table 2:** Percentage (with 95% CIs) of researchers in psychology, ecology and evolution who reported having used each Questionable Research Practice at least once. n = 555–626.

Questionable Research Practice	Psychology Italy Agnoli et al. [16]	Psychology USA John et al. [17]	Ecology	Evolution
Not reporting response (outcome) variables that failed to reach statistical significance#	47.9 *(41*.*3–54*.*6)*	63.4 *(59*.*1–67*.*7)*	64.1 *(59*.*1–68*.*9)*	63.7 *(57*.*2–69*.*7)*
Collecting more data after inspecting whether the results are statistically significant#	53.2 *(46*.*6–59*.*7)*	55.9 *(51*.*5–60*.*3)*	36.9 *(32*.*4–42*.*0)*	50.7 *(43*.*9–57*.*6)*
Rounding-off a p value or other quantity to meet a pre-specified threshold#	22.2 *(16*.*7–27*.*7)*	22.0 *(18*.*4–25*.*7)*	27.3 *(23*.*1–32*.*0)*	17.5 *(13*.*1–23*.*0)*
Deciding to exclude data points after first checking the impact on statistical significance	39.7 *(33*.*3–46*.*2)*	38.2 *(33*.*9–42*.*6)*	24.0 *(19*.*9–28*.*6)*	23.9 *(18*.*5–30*.*2)*
Reporting an unexpected finding as having been predicted from the start#	37.4 *(31*.*0–43*.*9)*	27.0 *(23*.*1–30*.*9)*	48.5 *(43*.*6–53*.*6)*	54.2 *(47*.*7–60*.*6)*
Filling in missing data points without identifying those data as simulated*	2.3 *(0*.*3–4*.*2)*	0.6 *(0*.*0–1*.*3)*	4.5 *(2*.*8–7*.*1)*	2.0 *(0*.*8–5*.*1)*

#note that these statements began with “in a paper,” in John et al. [17] and Agnoli et al [16].

*note that this was referred to as “falsifying data” in John et al. [17] and Agnoli et al [16] which may have influenced the difference in response rates.

## Results

Overall, researchers in ecology and evolution reported high levels of Questionable Research Practices (Table 2, Fig 1). However, the frequency with which researchers reported using these regularly was much lower (Fig 2) and qualitative analyses reveals use of these practices in ways that may be less questionable (S2 Supplementary Material).

### Comparing ecology, evolution and psychology researchers

The responses for ecology and evolution researchers were broadly similar to those from the samples of psychologists studied by John et al. [17] and Agnoli et al [16] (Table 2). One exception to this is that ecologists were less likely than psychologists or evolution researchers to report ‘collecting more data after inspecting whether the results are statistically significant’ (see also Fig 1). Both ecology and evolution researchers were also less likely to report excluding data points after checking significance than psychologists. On the other hand, both ecology and evolution researchers were more likely to acknowledge reporting an unexpected finding as expected than both samples of psychologists.

### Self-reported QRP use compared to expected QRP use amongst colleagues

Broadly, researchers’ self-reported QRP use was closely related to their estimates of prevalence of QRPs in the scientific community (Fig 1). However, in the case of QRPs 2, 5, 6, 9 and 10, expected prevalence was substantially higher than individual self-reported use, suggesting that these may be considered the least socially acceptable QRPs in the set.

### Frequency of individual researchers’ QRP use

It was extremely rare for researchers to report high frequency (‘frequently’, ‘almost always’) use of QRPs. Most reported usage was at low frequency (‘once’, ‘occasionally’), with many researchers reporting they had never engaged in these practices (Fig 2).

Age and career stage were not strong predictors of how frequently researchers used Questionable Research Practices (Kendall’s Tau of 0.05, 95% CI = 0.001–0.069 and 0.04, 95% CI 0.011–0.058 respectively) but there was a considerable correlation between how often participants thought the practice should be used and how often they used it (Kendall’s Tau = 0.6, 95% CI = 0.61–0.65). Those who used practices frequently or almost always were much more likely to indicate that they should be used often.

### Perceptions of scientific integrity

Researchers in ecology and evolution expressed considerable doubts about their community’s scientific integrity (Table 3), mostly in relation to QRPs rather than scientific misconduct. Concern about the integrity of researchers at their own institution was roughly equal to concern about the integrity of other institutions, nor was there any notable difference in concern about graduate students compared to senior colleagues or collaborators. Our participants expressed least concern about their own integrity, but 44.6% still indicated doubts over their own use of QRPs.

**Table 3:** Proportion (with 95% CI) of researchers in ecology and evolution (combined) who reported having doubts about scientific integrity*.

	Questionable Research Practices			Scientific Misconduct		
	Never	Once or Twice	Often	Never	Once or Twice	Often
Researchers from other institutions	8.9	56.6	34.5	39.0	55.5	5.5
	*(6*.*8–11*.*6)*	*(52*.*3–60*.*7)*	*(30*.*6–38*.*6)*	*(34*.*9–43*.*4)*	*(51*.*0–59*.*8)*	*(3*.*8–7*.*8)*
Research at your institution	27.9	52.2	20.0	69.2	29.1	1.6
	*(24*.*2–31*.*8)*	*(47*.*9–56*.*4)*	*(16*.*8–23*.*6)*	*(65*.*0–73*.*1)*	*(25*.*3–33*.*3)*	*(0*.*8–3*.*1)*
Graduate student research at your institution	31.0	48.6	20.4	72.5	25.6	1.8
	*(27*.*2–35*.*1)*	*(44*.*3–52*.*8)*	*(17*.*1–24*.*0)*	*(68*.*4–76*.*3)*	*(21*.*9–29*.*7)*	*(1*.*0–3*.*5)*
Senior colleagues or collaborators	31.5	50.8	17.7	73.3	24.7	2.0
	*(27*.*6–35*.*5)*	*(46*.*6–55*.*1)*	*(14*.*7–21*.*2)*	*(69*.*2–77*.*0)*	*(21*.*1–28*.*7)*	*(1*.*1–3*.*7)*
Your own research	52.2	44.6	3.2	97.9	2.0	0.0
	*(48*.*0–56*.*4)*	*(40*.*5–48*.*8)*	*(2*.*0–5*.*0)*	*(96*.*2–98*.*8)*	*(1*.*1–3*.*7)*	*(0*.*0–0*.*8)*

*note that not all researchers answered each component of the table above so the total sample size for each of the cells differs slightly, ranging from 488 to 539 samples per cell

### Qualitative data analysis

At the end of each QRP question, researchers had the opportunity to make additional comments on the practice. Overall, we were surprised by the proportion of researchers who made comments. For some QRPs half the researchers left comments, and often substantial ones. Here we have summarised the ecology and evolution groups’ comments together, having not detected any major differences between the groups in a qualitative assessment. We interpret the volume of additional comments positively, as evidence of a research community highly engaged with issues of research practice and scientific integrity.

The most frequently offered justifications for engaging in QRPs were: publication bias; pressure to publish; and the desire to present a neat, coherent narrative (Table 4). A full description of the qualitative analysis is available in S2 Supplementary Material.

**Table 4:** Frequently offered arguments against and justifications for various Questionable Research Practices, summarising qualitative comments provided by ecology and evolution researchers. Columns relate to the description of the questionable research practice, complaints respondents made about the practice, indications on why they thought that practice might be tempting, and conditions that respondents identified as justifying the practice.

**Cherry-picking**			
Description	Complaints	Temptation	Justifications
QRP 1: Not reporting studies or variables that failed to reach statistical significance *(n = 408)* QRP 2: Not reporting covariates that failed to reach statistical significance *(n = 350)* QRP 4: Reporting a subset of statistical models as the complete tested *(n = 386)*	- increases false positive rate - leads to redundant investigation - impedes interpretation - skews meta-analyses - there is important information in non-significant results - it is unethical	- hard to publish non-significant results - journal word limits - difficult to create a compelling story with non-significant results - complete report makes boring methods and result sections - running extra models improves understanding of the system	- original method was flawed - analyses were exploratory - results from multiple analyses were the same - they were excluded during formal model selection - variables correlated - data did not match model assumptions
“Sometimes lots of data are collected and tested. Often non-significant variables are thrown out if they're not integral to the story. I think this is okay.” “Not reporting non-significant results biases the big picture (e.g. meta-analysis), mislead other researchers into thinking that a question is unexplored . . .This publication bias however, is obviously a result of the publication system.” “If multiple model sets are tested they should all be presented, otherwise we risk presenting misleading results by trying a bunch of stuff until one turns out to be significant”			
**HARKing**			
Description	Complaints	Temptation	Justifications
QRP 3: Reporting an unexpected finding as having been predicted *(n = 371)*	- it is unethical - unexpected results need to be confirmed - increases false positive rate	- makes article sexier - reviewers ask for this - pressure to publish - not always clear exactly what was hypothesised	- new hypotheses arise from better understanding of the system - researchers can explain the result - researchers should have hypothesised something else
“well, this is a difficult one—in the statistical sense, this should not happen, but in current times scientists are forced to market their work as best as possible and this is one way to make it more publishable.” “Encourages, just-so stories, we can always come up with a suitable explanation and prediction. The key point here is to avoid doing so without noticing.” “I believe it should not be used but editors and reviewers often demand that exploratory results are framed as a priori hypotheses”			
**P-hacking**			
Description	Complaints	Temptation	Justifications
QRP 5: Rounding- off a p value or other quantity to meet a pre-specified threshold *(n = 409)* QRP 6: Deciding to exclude data points after first checking the impact on statistical significance *(n = 334)* QRP 7: Collecting more data for a study after first inspecting whether the results are statistically significant *(n = 364)* QRP 8: Changing to another type of statistical analysis after the analysis initially chosen failed to reach statistical significance *(n = 346)*	- it is unethical - increases false positive rate	- the 0.05 threshold is arbitrary anyway - hindsight bias - pressure to publish - reviewers may ask for more data or different analyses	- all results are presented - process is reported - decision not based on significance - additional data collection already planned - original analysis was poorly chosen - data didn’t meet assumptions of original analysis - new analysis better reflects ecological context - tests are conducted to test robustness of result
“Attempts to conform to strict cut-off significance thresholds demonstrate an adherence to conventional practice over understanding of probability (e.g. the difference between p = 0.013 and 0.010 is and should be viewed as trivial).” “This practice leads to statistical significance overshadowing effect sizes and biological significance.” “Again, one needs to be ethical. Science is about testing hypotheses with experiment, not about publishing p<0.05 in the sexiest journal possible. A priori and post priori hypotheses are both acceptable, but they need to be labelled as such.”			

## Discussion

Our results indicate that QRPs are broadly as common in ecology and evolution research as they are in psychology. Of the 807 researchers in our sample, 64% reported cherry picking statistically significant results in at least one publication; 42% reported *p* hacking by collecting more data after first checking the statistical significance of results, and 51% acknowledged reporting an unexpected finding as though it had been hypothesised from the start (HARKing). That these are similar to QRP rates in psychology is hardly surprising given that publication bias and the same publish-or-perish culture persists across disciplines. However, it is important to establish the QRP rate in ecology and evolution, as it provides important evidence on which to base initiatives to improve research practices in these disciplines.

### Disciplinary differences

Our results are most marked by how similar rates of QRPs were across disciplines, but a couple of differences are worth noting. Ecology researchers were less likely to report ‘collecting more data after inspecting whether the results are statistically significant’ (QRP7) than evolution researchers or psychologists. We suspect this reflects a difference in the constraints of field versus laboratory research, rather than differences in the integrity of the researchers. It is often not physically possible collect more data after the fact in ecology (field sites may be distant, available sites and budgets may be exhausted). This interpretation seems supported by evidence that many ecologists who stated that they had ‘never’ engaged in this practice indicated that they found it acceptable.

The first nine of the QRPs we asked about were certainly controversial practices, generating mixed responses. The tenth is qualitatively different; it essentially asks about data fabrication. The social unacceptability of this practice is well recognised, and we might therefore expect under reporting even in an anonymous survey. The comments volunteered by participants largely reflected this, for example “Is that the science of ‘alternative facts’?” and “It is serious scientific misconduct to report results that were not observed”. The proportion of researchers admitting to this was relatively high in ecology (4.5%) compared to evolution (2.0%), US psychology (2.3%) and Italian psychology (0.6%). However, it’s important to note that our wording of this question was quite different to that in the John et al and Agnoli et al surveys. They asked directly about ‘falsifying data’ whereas we asked a softer, less direct question about ‘filling in missing data points without identifying those data as simulated’. Fiedler et al (2015) found that modified question wording changed QRP reporting rates and we suspect our change to the wording has resulted in an elevated reporting rate. We will not speculate further about ecology researchers reporting a higher rate of this than evolution researchers because the numbers of researchers admitting to this action are very small in both groups and the 95%CIs on these proportions overlap considerably.

### Novel insights into the usage of QRPs

Our results contribute to the broader understanding of researchers’ practices in two important ways. First, our results on reported frequency provide new insight into the regularity with which researchers engage in these practices; previous surveys in psychology did not elicit this information and asked only if the practice had been used ‘at least once’. Information about frequency of use allows us to better estimate the disruption these practices may have had on the published literature. We show that while reports of having engaged in QRPs at least once are alarmingly high, virtually no researchers acknowledge using any of the QRPs more than ‘occasionally’. Secondly, our qualitative results offer new understanding of the perceived acceptability of these practices, and common justifications of their use.

Our qualitative analysis highlights the perception of a detrimental influence of the current publish-or-perish culture and rigid format currently required in many ecology and evolution journals. Researchers’ comments revealed that they feel pressure to present a short, cohesive story with statistically significant results that confirm a priori hypotheses, rather than a full (and likely messy) account of the research as it was conceptualised and conducted.

Researchers’ qualitative comments also drew attention to grey areas, where the distinction between QRPs and acceptable practice was less clear. For example, in many ecology and evolution articles no hypotheses are overtly stated but the way the background material is described in the introduction can imply that the result was expected; does this constitute HARKing? Similarly, a number of participants answering QRP 6 stated that, although they had technically changed models after investigating statistical significance, their decision to change models was based on finding an error in the original model or discovering that the data did not match the model assumptions. These participants are recorded as using this QRP but whether or not it was ‘questionable’ in their case is unclear.

### Social acceptability of QRPs

Discrepancies between individual researchers’ self-identified QRP use and their estimates of others’ use suggest that certain practices are less socially acceptable. When average estimates of others’ use are much higher than average self-report of the practice, it suggests that the practice is particularly socially undesirable and that self-report measures may underestimate prevalence [17]. In our results, the greatest discrepancies were observed for QRPs 2, 5, 6, 9, and 10 (see Fig 2), suggesting that self-reported prevalence may underestimate the true prevalence of these practices. In contrast, where there is little discrepancy between these two measures we can infer that the practice has gained a degree of social acceptability, for example QRPs 1, 4, 7, 8. These may be harder practices to shift, as researchers may not recognise them as problematic.

### Limitations of the current work

Some key limitations need to be considered when interpreting the results from our study. Firstly, our sample of ecology and evolution researchers might be biased. We contacted only researchers who had published in high impact factor journals, which pre-determined some demographics of our sample. For example, it likely limited the number of graduate students (6%). Our results should be understood as reflecting the practices of post-doctoral, midcareer and senior academic researchers almost exclusively. There is also very likely to be a self-selection bias in our sample of survey respondents. Those who are more confident in their practices–and perhaps more quantitatively confident in general–may have been more likely to respond. If this is the case, then it seems most likely that it would result in an underestimate of QRP rates in the broader ecology and evolution community rather than an overestimate.

Another limitation in the data set is that, in order to assure participants of their anonymity, we did not collect any data on their country of origin. Evidence from Agnoli et al [16] and John et al. [17] suggests that QRPs may be more prevalent among psychology researchers from some countries than others. It seems highly likely that the same is true among ecology and evolution researchers but without data on this we cannot speculate further.

Lastly, we collected survey responses between November 2016 and July 2017, it is theoretically possible that the rate of certain QRPs has changed over this time. However, as we ask participants whether they have used any of these practices “never”, “once”, “occasionally”, “frequently”, or “almost always”, we suspect that any behaviour changes in this time period will not be evident in responses to our surveys.

### Solutions

Our results indicate that there is substantial room to improve research practices in ecology and evolution. However, none of these problems are insurmountable. In fact, the correlation we found between acceptability and prevalence of QRPs and the justifications people provided in text (S2 Supplementary Material) suggest that the prevalence of these practices could be reduced by educating researchers about their ramifications. These practices are driven by a publish-or-perish research culture that puts emphasis on producing sexy, novel stories over solid science. The researchers in our sample often commented on this; one researcher commented “the absence of significant result is so hard to publish that people (me included) finally don't even try to publish it. The absence of negative result in publications doesn't indicate that it wasn't tested, only that editors don't select them for publication, because they consider them not enough sexy” and another researcher stated “I think there is a problem in ecology where the 'sexy' findings are promoted, and they often find their way into high ranking journals. Other solid pieces of work often languish in 'specialist' journals”. This culture is perpetuated by science’s reliance on publishers which, as private companies, may be more concerned with their reputation and finances than with furthering the scientific endeavour. The open science movement has given rise to a series of solutions that help reduce the temptation for and prevalence of these QRPs [6,11,13,36]. A number of these solutions rely on changes in journal practices or institutional policies that may be difficult to implement because these interventions may (at least in the short term) meet resistance from publishing companies. Even though current incentive structures may favour the status quo in scientific publishing, researchers have made inroads through combinations of individual and coordinated action A promising tool for individual researchers to adopt is preregistration. A thorough preregistration specifies researchers’ hypotheses, how they will decide on their sample size, data exclusion criteria, and the analyses they will conduct, among other things. This helps researchers think their research through thoroughly, improving its rigor, as well as protecting against HARKing, cherry-picking and p-hacking [37,38]. Despite the growing use of pre-registration, its widespread adoption remains uncertain in ecology and evolutionary biology. Obstacles include resistance from researchers who mistakenly believe that preregistration limits their creativity and ability to conduct exploratory work [37], but also from journals that still preferentially accept manuscript with ‘positive’ results and clear stories, thus incentivizing HARKing and other QRPs. However, many journals are published by scientific societies and have editorial boards populated by practicing scientists, and these individual scientists can work to promote favourable practices. Changes to reduce QRPs can happen, but this movement is still young.

Some editors in ecology and evolutionary biology have also instigated important changes such as requiring data archiving at a handful of prominent journals [39]. Although there has been some limited push-back against data archiving [40] and compliance falls far short of perfect [41], this case demonstrates the potential impact of committed people in influential positions. The archiving movement is now spreading beyond data [42], and a small but growing number of journals are starting to use rigorous checklists for authors to encourage more transparent reporting of important aspects of methods and results (e.g. *Conservation Biology*, *Nature*).

## Conclusion

The use of Questionable Research Practices in ecology and evolution research is high enough to be of concern. The rates of QRPs found in our sample of 807 ecologists and evolutionary biologists are similar to those that have been found in psychology, where the reproducibility rates of published research have been systematically studied and found to be low (36–47% depending on the measure [2]). Researchers in our survey offered justifications for their practices including: publication bias; pressure to publish; and the desire to present a neat, coherent narrative. We recommend that all journals in ecology and evolution adopt editing and reviewing checklists to ensure more complete and transparent reporting, encourage preregistration and registered reports article formats to minimise HARKing, and encourage open code and data whenever possible.

## Supporting information

S1 Supplementary Material Questionable research practice survey. (PDF)

S2 Supplementary Material Qualitative data analysis. (DOCX)

S3 Supplementary Material Disambiguating QRP 1 cherry-picking vs the file drawer. (DOCX)
