# Supplementary material for: Questionable research practices in ecology and evolution
Source: PLoS One. 2018 Jul 16;13(7):e0200303. doi: 10.1371/journal.pone.0200303 (PMC6047784; doi:10.1371/journal.pone.0200303)
Supplement: S1 Supplementary Material — (PDF) [file pone.0200303.s001.pdf]

Plain Language Statement

Traditional Research Practices Survey

We are studying research practices in ecology. You have been invited to participate in this survey because you have published work in a high-impact ecology journal.

If you agree to participate, you will be presented with a list of 10 traditional study design and analysis practices, and asked to:
a) estimate the percentage of researchers in your field who engage in each practice,
b) indicate whether you have ever engaged in a practice,
c) give your opinion on this practice.

We expect this survey to take less than 15 minutes. At the end you can choose to be emailed the results of this survey.

Participant Information

Your answers will be stored securely and anonymously. Overall results may be published, but nothing that could identify any individual. The processed data, without any identifying information, will be stored on the Open Science Framework website. Your participation will help us better understand how ecologists work. You can stop the survey at any time.

Ethics clearance

This research project has been approved by the Human Research Ethics Committee of The University of Melbourne. (Ethics ID number: 1646917.1) If you have any concerns or complaints about the conduct of this research project, which you do not wish to discuss with the research team, you should contact the Manager, Human Research Ethics, Office for Research Ethics and Integrity, University of Melbourne, VIC 3010. Tel: +61 3 8344 2073 or Fax: +61 3 9347 6739 or Email: HumanEthics-complaints@unimelb.edu.au. All complaints will be treated confidentially. In any correspondence please provide the name of the research team or the name or ethics ID number of the research project.

This research is funded by an Australian Research Council Future Fellowship. ID: FT150100297.

Researchers

Dr Fiona Fidler (Principal Investigator, The University of Melbourne) (fidlerfm@unimelb.edu.au ph: +61 3 9035 6315). Dr Shinichi Nakagawa (The University of New South Wales). Dr Tim Parker (Whitman College). Mr Ashley Barnett (The University of Melbourne).

Consent

Informed consent is implied by entering the survey. The data you anonymously provide may be published in peer reviewed papers and presented at academic conferences. Neither your name or any other identifying information is collected. You may stop the survey at any time.

Not reporting studies or variables that failed to reach statistical significance (e.g. $p \leq 0.05$) or some other desired statistical threshold.

a) Please estimate the percentage of research ecologists who you believe **have engaged** in this practice on at least one occasion:

0 10 20 30 40 50 60 70 80 90 100

b) Have you ever engaged in this practice?

Never

Once

Occasionally

Frequently

Almost Always

c) What's your opinion of this practice?

It should be used almost always

It should be used often

It should only be used rarely

It should never be used

d) *Optional Question:* Why do you think this practice should or shouldn't be used?

Not reporting covariates that failed to reach statistical significance (e.g. p ≤ 0.05) or some other desired statistical threshold.

a) Please estimate the percentage of research ecologists who you believe **have engaged** in this practice on at least one occasion:

0 10 20 30 40 50 60 70 80 90 100

b) Have you ever engaged in this practice?

Never
Once
Occasionally
Frequently
Almost Always

c) What's your opinion of this practice?

It should be used almost always
It should be used often
It should only be used rarely
It should never be used

d) *Optional Question:* Why do you think this practice should or shouldn't be used?

Reporting an unexpected finding or a result from exploratory analysis as having been predicted from the start.

a) Please estimate the percentage of research ecologists who you believe **have engaged** in this practice on at least one occasion:

0 10 20 30 40 50 60 70 80 90 100

b) Have you ever engaged in this practice?

Never

Once

Occasionally

Frequently

Almost Always

c) What's your opinion of this practice?

It should be used almost always

It should be used often

It should only be used rarely

It should never be used

d) *Optional Question:* Why do you think this practice should or shouldn't be used?

Reporting a set of statistical models as the complete tested set when other candidate models were also tested.

a) Please estimate the percentage of research ecologists who you believe **have engaged** in this practice on at least one occasion:

0 10 20 30 40 50 60 70 80 90 100

b) Have you ever engaged in this practice?

Never

Once

Occasionally

Frequently

Almost Always

c) What's your opinion of this practice?

It should be used almost always

It should be used often

It should only be used rarely

It should never be used

d) *Optional Question:* Why do you think this practice should or shouldn't be used?

Rounding-off a p value or other quantity to meet a pre-specified threshold (e.g., reporting p = 0.054 as p = 0.05 or p = 0.013 as p = 0.01).

a) Please estimate the percentage of research ecologists who you believe **have engaged** in this practice on at least one occasion:

0 10 20 30 40 50 60 70 80 90 100

b) Have you ever engaged in this practice?

Never

Once

Occasionally

Frequently

Almost Always

c) What's your opinion of this practice?

It should be used almost always

It should be used often

It should only be used rarely

It should never be used

d) *Optional Question:* Why do you think this practice should or shouldn't be used?

Deciding to exclude data points after first checking the impact on statistical significance (e.g. $p \leq 0.05$) or some other desired statistical threshold.

a) Please estimate the percentage of research ecologists who you believe **have engaged** in this practice on at least one occasion:

0 10 20 30 40 50 60 70 80 90 100

b) Have you ever engaged in this practice?

Never

Once

Occasionally

Frequently

Almost Always

c) What's your opinion of this practice?

It should be used almost always

It should be used often

It should only be used rarely

It should never be used

d) *Optional Question:* Why do you think this practice should or shouldn't be used?

Collecting more data for a study after first inspecting whether the results are statistically significant (e.g. $p \leq 0.05$).

a) Please estimate the percentage of research ecologists who you believe **have engaged** in this practice on at least one occasion:

0 10 20 30 40 50 60 70 80 90 100

b) Have you ever engaged in this practice?

Never

Once

Occasionally

Frequently

Almost Always

c) What's your opinion of this practice?

It should be used almost always

It should be used often

It should only be used rarely

It should never be used

d) *Optional Question:* Why do you think this practice should or shouldn't be used?

Changing to another type of statistical analysis after the analysis initially chosen failed to reach statistical significance (e.g. $p \leq 0.05$) or some other desired statistical threshold.

a) Please estimate the percentage of research ecologists who you believe **have engaged** in this practice on at least one occasion:

0 10 20 30 40 50 60 70 80 90 100

b) Have you ever engaged in this practice?

Never

Once

Occasionally

Frequently

Almost Always

c) What's your opinion of this practice?

It should be used almost always

It should be used often

It should only be used rarely

It should never be used

d) *Optional Question:* Why do you think this practice should or shouldn't be used?

Not disclosing known problems in the method and analysis, or problems with the data quality, that potentially impact conclusions.

a) Please estimate the percent of research ecologists that **have engaged** in this practice on at least one occasion:

0	10	20	30	40	50	60	70	80	90	100

b) Have you ever engaged in this practice?

Never

Once

Occasionally

Frequently

Almost Always

c) What's your opinion of this practice?

It should be used almost always

It should be used often

It should only be used rarely

It should never be used

d) *Optional Question:* Why do you think this practice should or shouldn't be used?

Filling in missing data points without identifying those data as simulated.

a) Please estimate the percentage of research ecologists who you believe **have engaged** in this practice on at least one occasion:

0 10 20 30 40 50 60 70 80 90 100

b) Have you ever engaged in this practice?

Never

Once

Occasionally

Frequently

Almost Always

c) What's your opinion of this practice?

It should be used almost always

It should be used often

It should only be used rarely

It should never be used

d) *Optional Question:* Why do you think this practice should or shouldn't be used?

Researcher Integrity

Have you ever had doubts about the scientific integrity of research in ecology? *If this questions doesn't display properly on your device, please skip to the next question.*

	Mild issues/Questionable Research Practices			Serious issues/Scientific Misconduct		
	Never	Once or Twice	Often	Never	Once or Twice	Often
Research from other institutions	○	○	○	○	○	○
Research at your institution	○	○	○	○	○	○
Graduate student research at your institution	○	○	○	○	○	○
Senior colleagues and/or collaborators	○	○	○	○	○	○
Your own research	○	○	○	○	○	○

Demographic Info

Are you a... ?

Graduate student

Post doctoral fellow

Mid-career research fellow/academic

Senior research fellow/academic

What is your gender?

Male

Female

Non-binary/ third gender

Prefer to self-describe

Prefer not to say

What is your age?

Under 20

20-29

30-39

40-49

50-59

60-69

70+

What is your sub-discipline?

Thank you for participating. Before you leave, do you have any comments, questions, or suggestions about this survey? If so, please enter them below.

Powered by Qualtrics
