# Supplementary material for: Questionable research practices in ecology and evolution
Source: PLoS One. 2018 Jul 16;13(7):e0200303. doi: 10.1371/journal.pone.0200303 (PMC6047784; doi:10.1371/journal.pone.0200303)
Supplement: S2 Supplementary Material — (DOCX) [file pone.0200303.s002.docx]

# Supplementary Material S2: Qualitative Data Analysis

QRP1: Not reporting response (outcome) variables that failed to reach statistical significance

Roughly half (51%, 408/807) of the researchers in our survey added additional open-ended comments in their response to QRP1. Many responses highlighted ambiguity in the question, with two main interpretations (and combinations of these interpretations) of the question appearing in their comments:

1. failure to discuss all statistical tests in a manuscript, i.e., cherry-picking (n=232)
2. failure to publish all studies regardless of statistical significance, i.e., file-drawer problem (n=107; n=34 mentioned both 1 and 2)

In the supplementary material we present a further break down QRP rates for each of these interpretations. Here we describe in more detail comments elicited; despite the different interpretations, many similar themes emerged.

*Cherry-picking*

**Indicative quotes:**

Pro cherry-picking: “I think this is part of the publishing problem: there isn't enough space to talk about everything that you did, particularly what proved not to be an important effect”

Anti cherry-picking: “The more tests you perform the higher your chances of getting significant results by chance. Also negative results may also be interesting”

Mixed opinions of cherry picking: “Ideally, everything should be reported, including exploratory analyses. But this is practically impossible.”

Researchers drew most attention to: the difficulty of getting an article with any non-significant results published (n=32); constraints imposed by word limits that limit full reporting (n=39); difficulty in creating a compelling story around non-significant results (n=24).

The most frequently identified circumstances under which a case for justified cherry-picking were: if the analysis was exploratory or post-hoc and failed to reach statistical significance (n=28); if a flaw was discovered in the original method and so it was not reported (n=12); if the study was a preliminary or pilot study and failed to reach statistical significance (n=2); if other analyses were attempted but they did not change the interpretation of results and so were not reported (n=1). A small number expressed that cherry picking was necessary for choosing which elements to include in a coherent story (n=5) and that reporting everything would ‘flood’ the literature with non-significant findings (n=3).

In contrast, others commented that: there was always important information in non-significant results (n=19) and explicitly expressed understanding and concern that cherry-picking increases type I error rate (n=8); leads to redundant investigations (n=6); and impedes readers’ interpretation of the results (n=8). Some suggested that additional tests could be mentioned but not discussed in detail (n=9), or present the details in supplementary materials (n=12).

*File drawer problem*

**Indicative quotes:**

Pro file drawer problem: “If there is no significant effect, and the study did not have sufficient power to detect a meaningful difference, then the paper would be hard to publish anyway, and there may be little value in publishing it.”

Anti file drawer problem: “It is the significance or reporting bias that even has to be corrected for in meta-analyses. Non-significant studies are less submitted, because less published.”

Mixed opinion of file drawer problem: “I think it is sometimes inevitable when exploring complex datasets ; it is frequent to investigate the influence of some variables that are not crucial to the main question at the exploratory stage, and to omit it from the final manuscript eventually. I believe banishing such a practice would be very difficult. Given the level of energy required to publish, it would be difficult to publish all the student projects that have not been fully completed, for example”

Amongst researchers referring to the file drawer problem, many mentioned publication bias/difficulties in publishing statistically non-significant results (n=50) and suggested that, while not ideal, many researchers prioritise writing up significant results to maximise high profile, well cited publications (n=37). Some added that relegating studies to the file drawer may be justified if that study was low powered or methodologically flawed (n=18) but this was tempered by concerns that there is usually important information in non-significant results (n=9), and that failure to publish negative results skews meta-analysis (n=7), and leads to redundant investigations (n=5).

QRP 2: Not reporting covariates that failed to reach statistical significance

**Indicative quotes:**

Pro QRP 2: “If there is substantial space saving or simplicity in leaving the non-significant information out, especially in exploratory analyses, readers don't need to be burdened with the non-essential information”

Anti QRP 2: “If there is a reason to believe that a covariate is biologically significant, but it is not statistically significant, the result should still be reported. Failing to report negative results can lead researchers to waste time searching for effects that aren't there.”

Mixed opinion of QRP 2: “this practice should be avoided, but we all know that it is easier to present significant results so we always select the best (more meaningful) variables”

43% (350/807) researchers made additional comments on their response to QRP2. Again, many mentioned the difficulty of publishing papers including non-significant results (n=11), and pressures associated with writing brief articles/word limits (n=34) with compelling stories (n=11), all of which present obstacles to complete reporting of all tested covariates.

The most frequently identified circumstances under which a case for justified failure to report covariates were: the statistically non-significant covariates were detected in a pilot, exploratory or post-hoc tests (n=30); not specified in the original hypothesis (n=28) or included in the final model (n=13) and/or excluded during model selection (n=9); not ‘meaningful’ variables (n=21); problematic, correlated or confounded (n=16). Some stipulated that the above justifications only apply if the process was explicitly described in the resulting paper (n=18).

Conversely, many comments proposed reasons to avoid using this practice arguing that: there is important information in non-significant results (n=40); not reporting all covariates distorts meta-analysis (n=14); inflates the Type I error rate (n=18) and impedes the interpretation of results (n=9).

Some researchers took the opportunity to provide suggestions for how to include information about covariates without compromising the length or readability of manuscripts (n=43) and the highlighted that importance of their inclusion in supplementary material, if excluded from the final write-up (n=23). Finally, within their comments on this QRP, some researchers questioned the usefulness of using p values in science (n=5) and expressed concern about arbitrary thresholds (e.g., p<0.05) (n=2).

QRP 3: Reporting an unexpected finding as having been predicted from the start

**Indicative quotes:**

Pro QRP 3: “It helps highlight a specific point/message”

Anti QRP 3: “This is dishonest and cannot be tolerated in the scientific community”

Mixed opinion of QRP 3: “This practice helps to make the text easier to follow, although I agree that this may introduce some bias.”

46% (371/807) researchers made additional comments on their response to QRP3. The comments on this QRP were more polarised than for QRPs 1 and 2. Some researchers stated that it should be acceptable to report unexpected results as such (n=28) and/or that they have been specifically told to engage in HARKing, e.g., report different hypotheses in line with their results by editors, reviewers or senior collaborators (n=19). Comments in support of this practice mentioned that it improves how sexy or publishable the story is (n=97) and there is pressure to publish prolifically (n=44).

The most frequently identified circumstances under which HARKing was considered justified included: rewriting hypotheses if the results could be explained by something that the researcher should have known from the start but didn’t (n=14); a new hypotheses arose from gaining a better understanding of the study system through the research conducted (n=12); the new hypothesis was still close to the original (n=3); or simply because the researchers were able to generate an explanation for unpredicted/exploratory results (n=5). Others felt that original hypotheses were not important because what researchers expect should not matter; results should speak for themselves (n=12).

Those opposed to the practice focused on ethics, with a large number of comments (n= 97) referring to honesty, ethics, deception and right vs wrong scientific practice. Some explained the need for unexpected results need to be confirmed in follow up studies (n=19); that this practice risks increasing the Type I error rate (n=16) and the inappropriateness of testing new hypotheses with pre-existing data (n=6).

More nuanced comments expressed that HARKing had grown out of a fault in the current publication system. They described how the formulaic introduction – methods – results – discussion scientific article encouraged signposting all results, which easily translates into specifying hypotheses for all results.

Others comments focussed on the blurred line between good practice and HARKing (n=19). For example, they explained that many papers in ecology and evolution don’t include explicit hypotheses. Instead hypotheses are implied by the inclusion or exclusion of certain background literature. If one selects the background literature to fit with result outcomes, does that constitute HARKing? Similarly, others mentioned that hypotheses often aren’t recorded precisely before the research commences and this means that it becomes difficult to determine whether results were or were not expected (n=11), especially given hindsight bias (n=4).

QRP4: Reporting a subset of tested statistical models as if they were the complete tested set

**Indicative quotes:**

Pro QRP 4: “If different ways of analyzing the data give the same result, I don't see the need to report all the different ways”

Anti QRP 4: “Stating that you have presented the complete set of tested models when you have not is a falsehood”

Mixed opinion of QRP 4: “I think it is a byproduct of the work. Often you may not realise your model is quite right and have to go back and revisit it again. Often, it is helpful to use multiple analyses in order to examine if the final analysis is robust, and this would often be reported in the paper, without specific results from these models. Of course, this approach shouldn't be used in an unethical way to dredge data for significant results using different types of models.”

48% (386/807) of researchers made additional comments in their response to QRP4. Many expressed concern about the possibility for inflated Type I error rates (n=63) and the lack of honesty and transparency (n=32) associated with this practice.

A substantial number of comments mentioned the potential benefits of running multiple models: provides a greater understanding of the system (n=17); can be used to determine how robust the finding is to the exact specifications of the model (n=6); and that results could be used for multi-model inference (n=7). Many comments supported the practice of not reporting all models run. For example: researchers should use their discretion to determine which models to report (n=14); it is only necessary to present the final model (n=12); testing multiple models can allow you to rule out models that are not ecologically sensible (n=8) or informative (n=28) and those do not need reporting; it is ok to report only those models which perform best in terms of converging or meeting model assumptions (n=40), and models with errors (that forgot to include a term, used the wrong distribution for the data etc should not be reported (n=29), nor should those that produce very similar results (n=12). Further, many comments suggested that reporting all of the models would lead to unwieldy or lengthy methods sections which is undesirable for readers and journals (n=46). Some indicated that simply mentioning the other models was sufficient (n=22) or that they should be presented in supplementary material only (n=19)

One of the more subtle and telling themes emerging from the comments on QRP4 is the distinction researchers make between what is acceptable for exploratory vs confirmatory research. Many mentioned that it was acceptable to not report all models run if the research was exploratory (n=47). Similarly, others explicitly commented that models representing a priori hypotheses should always be reported (n=16).

QRP5: Rounding-off a p value or other quantity to meet a pre-specified threshold

**Indicative quotes:**

Pro QRP 5: “From my point of view 0.054 or 0.05 are basically the same and should be interpreted as marginally significant and it should be explained so in a paper. But I am ok with rounding in the presentations at conferences to avoid the smartasses that try someone just to put down.”

Anti QRP 5: “If a value is known to exceed a pre-specified threshold, then it should be treated as such. “

Mixed opinion of QRP 5: “I basically present 3SF most of the time. Sometimes that makes rounding errors in favour of significance; sometimes not.”

Roughly half (51%, 409/807) of researchers made additional comments in their response to QRP5. Many identified the practice was ‘wrong’ or ‘dishonest’ (n=89) and pointed out that it introduced bias to the literature (n=11). In contrast, others pointed out that because the 0.05 threshold is arbitrary (n=78) the practice was not a serious problem (n=52).

Some comments offered practical suggestions for how to report and interpret *p* values that would help overcome this QRP, including: reporting precise (e.g., p=.003) rather than relative (p<.05)  *p* values (n=74); always using the same reporting rule, e.g., reporting *p* to 3 decimal places, regardless of statistical significance (n=42); providing sufficient detail for readers to their own decision about statistical significance (n=38); and always reporting the relevant sample size (n=6). Others recommended focussing on effect size (n=19) and biological significance (n=13) instead of *p* values. Some participants noted that certain journals limit the number of decimal places that *p* values can be reported to which may lead researchers to employ this QRP (n=11).

Interestingly, there was a notable difference in comments expressing a Neyman-Pearson approach to *p* values (n=39 comments pointing out that that the <0.05 threshold means than 0.05 and 0.054 are both non-significant) and those taking a Fisherian approach (n=53 comments pointing out that there is little difference in strength of evidence between 0.05 and 0.054).

QRP6: Deciding to exclude data points after first checking the impact on statistical significance

**Indicative quotes:**

Pro QRP 6: “A careful examination of outliers, eventually followed by its exclusion of the analysis, is part of good practice in statistics. It should however be well documented.”

Anti QRP 6: “Excluding outliers is sometimes unavoidable, but it must not be based on the result, because this inflates Type I error rates and therefore leads to spurious results.”

Mixed opinion of QRP 6: “Excluding outliers can be a good practise if the detection of outliers has been performed before other stats and in case of experiments or field data, we are sure that the outliers correspond to abnormal individuals. But the exclusion of data searching for a significant results is just cheating the results removing the individuals which don't fit with our hypothesis. That is completely wrong.”

44% (354/807) of researchers made additional comments in their response to QRP6. Many comments expressed that this practice is unethical (n=65) and that it inflates type I error rate (n=22). A smaller number (n=9) suggested that analysis should always include results from the full raw data set. Many (n=38) asserted that it was important to make all decisions about excluding outliers before conducting statistical analyses; a few explicitly expressed concern about hindsight bias (n=4). A large number of comments (n=60) suggested that removing outliers of the basis of sensitivity analysis was acceptable, assuming both the full and trimmed datasets are provided.

A number of comments placed caveats on the general practice of removing outliers: that the procedure and rationale should always be explicitly documented (n=70); that removal should only be done according to specific procedures (n=25); should never be based on the statistical significance of the outcome (n=46); should only occur if there is a biological reason to remove them (n=52) or if they violate the model assumptions (n=11). Many researchers also pointed out that outliers themselves can often be caused by data collection or transcription errors (n=52).

QRP7: Collecting more data after inspecting whether the results are statistically significant

**Indicative quotes:**

Pro QRP 7: “As long as the experimental design is not altered as a result of preliminary trends observed during data collection, I see little harm in this practice. The process of collecting data is often a long one, and developing analysis scripts on incomplete datasets is a way to efficiently use time.”

Anti QRP 7: “If you collect more data in order to get a significant result, you are too motivated to ensure the data comes out the way you want.”

Mixed opinion on QRP 7: “Although this could be useful to prevent spending money and time in some cases, this should be the exception, not the rule.”

45% (364/807) of participants provided a written response to QRP7. Some comments provided reasons that the practice shouldn’t be used: that it increase the type I error rate (n=33), is dishonest (n=3), or that the sample size used in a study should be determined before data collection (n=38). However, they noted that there was pressure to use this practice because reviewers often request additional data collection (n=6), and there is a strong pressure to publish prolifically (n=6). A large number (n=31) of participants suggested that this practice would be infeasible for their work due to logistical or funding constraints. Similarly, many comments suggested that this practice can help effectively allocate resources (n=34), conducting preliminary analysis and deciding whether it is worthwhile investing funding in pursuing the question based on trends in the data.

Participants also raised circumstances in which this practice might be justified. Broadly these circumstances fell under two main headings 1) cases where more data collection was planned and people examined it early, and 2) cases where analyses result in adding more samples than initially planned. The former might involve analysing the results early for a conference presentation (n=3) or when collecting long-term datasets (n=6). Justifications of using this practice in the second case are more varied. Some comments suggest that: more data is always better (n=55), it is ok to do this if the initial data is collected as part of a pilot study (n=60), if it is used to conduct a power analysis to determine how many samples are required (n=49), if the original study is underpowered (n=58), if additional data collection is conducted carefully to minimise bias (n=8), or if the process is clearly described in text (n=21).

QRP8: Changing to another type of statistical analysis after the analysis initially chosen failed to reach statistical signiﬁcance (e.g. p ≤ 0.05) or some other desired statistical threshold

**Indicative quotes:**

Pro QRP 8: “If an analysis does not seem to show a significant difference, then it is not unreasonable to try another analysis type. So long as the analysis is validly applied, you can adjust to meet the needs of your data.”

Anti QRP 8: “Bad form - stats should be framed in advance. The sole exception might be if you are explicitly running an exploratory analysis, and don't have a knowledge base for your system of where to start. In any case though, that should be clearly reported (and I don't think I've ever seen a paper like this).”

Mixed opinion on QRP 8: “Again, if the intent is to keep trying to new tests until one is statistically significant, this is dishonest. If the intent is to use the correct test, and you realize that you have used the incorrect one after testing it, well, that's a more thorny issue. It does happen and in the real world, it is hard to avoid as we are not all experts in all the methods and learning about new methods happens "on the fly". I have done this "by accident" lots, but I strive to avoid it wherever possible.”

43% (347/807) of participants volunteered comments about this QRP. Participants expressed considerable concern that this practice increases the type I error rate (n=42), and is dishonest or unethical (n=21). Some comments expressed that statistical analyses should be decided a-priori (n=29) and only changed if the original analysis was poorly chosen (n=58). Many comments suggested that if the analysis methods are changed this should not be informed by statistical significance (n=61), but instead based on the match between the data and model assumptions (n=61), ecological relevance (n=11), or if researchers learn about a superior method (n=32). Counter to this, some comments suggested that it was justified to change analyses as long as the research is intended to be exploratory (n=26)

Others stated that changing analysis methods was justified if the methods were thoroughly reported in the manuscript (n=25), the results of the alternative tests were reported in the manuscript (n=30) or the supplementary material (n=3). This was considered particularly justified if multiple tests were run to test how robust the findings were across different statistical analyses (n=36). In a similar vein, iIt was common for participants to suggest that it was unlikely that different analyses would find an effect if the original analysis failed to find one (n=11).

QRP9: Not disclosing known problems in the method and analysis, or problems with the data quality, that potentially impact conclusion

**Indicative quotes:**

Pro QRP 9: “Almost all analyses in evolution have many potential pitfalls. I don't think it's necessary to discuss every one for every analysis, but rather just focus on the key ones that could qualitatively alter interpretation of results.”

Anti QRP 9: “A full disclosure of study weaknesses is one of the most important aspects of the discussion section of a research paper. It helps make more informed conclusions on often-noisy ecological data.”

Mixed opinion on QRP 9: “Although caveats can be distracting and dispiriting in a publication, it's important that any major sources of potential error or uncertainty are addressed. Limits of space mean that researchers do have to exercise their best judgement as to what potential problems are most likely to effect the conclusions, however.”

31% (252/807) of participants volunteered a comment on this QRP. Many comments suggested that this practice was dishonest and bad scientific practice (n=73). The practice was seen to be problematic because it limits readers’ ability to accurately interpret the results (n=36), results in unreproducible studies (n=7), and because it leads others to make the same mistakes (n=11). This is tempered by an acknowledgement that fully disclosing limitations may be precluded by journal word limits (n=24) and be less likely to result in a high impact publication (n=32). Some suggested that this may be overcome by only not mentioning the well-known problems with a particular methodology (n=12), focussing on the key limitations that are likely to impact the conclusions (n=34), and where necessary including the additional limitations in supplementary material (n=5).

Participants believed that neglecting to mention limitations will ‘come back to bite’ researchers in the future (n=7) and that the peer review process will protect against neglecting limitations (n=5). Peer review’s protective ability is particularly important as many comments suggested that limitations are often left out due to researcher ignorance rather than a deliberate decision to hide limitations (n=13)

QRP10: Filling in missing data points without identifying those data as simulated

**Indicative quotes:**

”I introduced some mean values to a couple of missing points in a survey study once and did not report it. Maybe I should have done it, but it was more that I forgot.”

“Many ecologists might impute missing data while saying that they do so, but not identifying exactly which data points are imputed. I don't have a big problem with this.”

“Fabricating data is definitely an ethical violation.”

30% (246/807) of participants made a comment on this questionable research practice. The responses to this practice were a lot more one-sided than for the other practices discussed, the vast majority of responses vehemently rejected the practice (n=172) e.g. “Who does this? This is essentially "faking" data!”. Many responses specifically suggested that this was fraud/fabrication/malpractice (n=47) or commented on how dishonest the practice is (n=24). Some participants noted that, imputing data can be acceptable but only if it is well described and thoroughly documented (n=67). Three participants suggested that they had used this practice. In one case this involved inserting mean values in the place of missing data points (quote above), in one case this involved re-simulating date for a simulation study when a reviewer wanted information on a statistic that hadn’t been originally recorded, the other instance was ambiguous “If you knew the truth ....”.
