# Supplementary material for: Questionable research practices in ecology and evolution
Source: PLoS One. 2018 Jul 16;13(7):e0200303. doi: 10.1371/journal.pone.0200303 (PMC6047784; doi:10.1371/journal.pone.0200303)
Supplement: S3 Supplementary Material — (DOCX) [file pone.0200303.s003.docx]

# Supplementary Material S3: Disambiguating QRP 1 Cherry-picking vs the File Drawer

As mentioned in our qualitative analysis, it was evident that people had interpreted QRP 1 in two different ways: either as referring to cherry-picking or the file drawer problem. These are two distinct problems, whereby keeping studies in the file drawer is seen as much more acceptable than cherry-picking (based on the tenor of qualitative responses). To investigate whether there these different interpretations are associated with differences in the results we evaluated QRP 1 as a whole (presented in the manuscript) but also two subsets, one with just those responses where qualitative data revealed that they had only considered cherry-picking,one with just those responses where qualitative data revealed that they had only considered the file drawer problem.

Figures S1-S3 show that the results for cherry-picking alone (Figure S2) are similar to those from the full dataset (Figure S1) but that participants who interpreted QRP to be about the file drawer problem were less likely to say that they never use the practice (Figure S3)


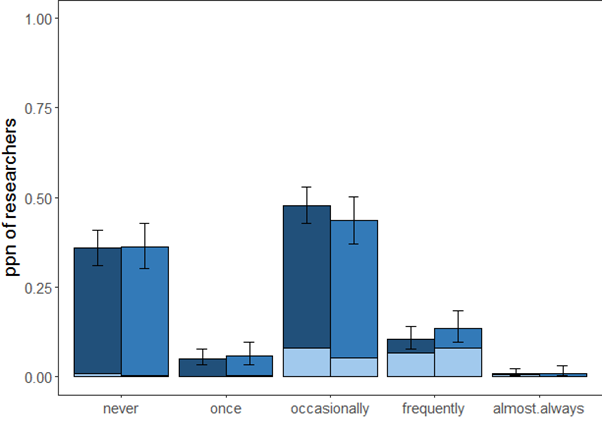


Figure S1: QRP 1 Results the full dataset as a comparison point (n=873)


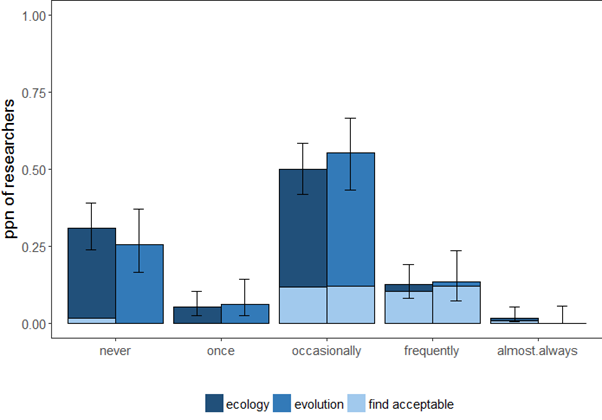


Figure S2: QRP 1 Results from participants exclusively considering cherry-picking (n=209)

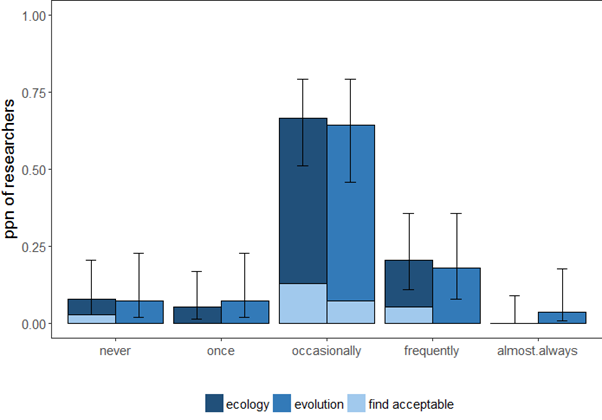


Figure S3: QRP 1 Results from participants exclusively considering the file drawer problem (n=71)
